# Hardening of Additive Manufactured 316L Stainless Steel by Using Bimodal Powder Containing Nanoscale Fraction

**DOI:** 10.3390/ma14010115

**Published:** 2020-12-29

**Authors:** Aleksandr M. Filimonov, Oleg A. Rogozin, Denis G. Firsov, Yulia O. Kuzminova, Semen N. Sergeev, Alexander P. Zhilyaev, Marat I. Lerner, Nikita E. Toropkov, Alexey P. Simonov, Ivan I. Binkov, Ilya V. Okulov, Iskander S. Akhatov, Stanislav A. Evlashin

**Affiliations:** 1 Center for Design, Manufacturing & Materials (CDMM), Skolkovo Institute of Science and Technology, 30 Bolshoy Boulevard Str., bld. 1, 121205 Moscow, Russia; Aleksandr.Filimonov@skoltech.ru (A.M.F.); O.Rogozin@skoltech.ru (O.A.R.); D.Firsov@skoltech.ru (D.G.F.); Yulia.Kuzminova@skoltech.ru (Y.O.K.); A.Simonov2@skoltech.ru (A.P.S.); I.Akhatov@skoltech.ru (I.S.A.); 2 Institute of Metals Superplasticity Problems of the Russian Academy of Sciences (IMSP), 39 Stepana Khalturina Str., 450001 Ufa, Russia; nikocem17@gmail.com (S.N.S.); alex.zhilyaev@hotmail.com (A.P.Z.); 3 Laboratory of Mechanics of Gradient Nanomaterials, Nosov Magnitogorsk State Technical University, 38 Lenin Str., 455000 Magnitogorsk, Russia; 4 Institute of Strength Physics and Materials Science of Siberian Branch of the Russian Academy of Sciences (ISPMS), 2/4 Akademicheskii pr., 634055 Tomsk, Russia; lerner@ispms.ru (M.I.L.); Zerogooff@gmail.com (N.E.T.); 5 Scientific and Educational Center “Additive Technologies”, National Research Tomsk State University, 36 Lenin Avenue, 634050 Tomsk, Russia; 6 Materials and technology, Bauman Moscow State Technical University, 2 Baumanskaya Str., bld. 5/1, 105005 Moscow, Russia; crockinline@yandex.com; 7 Faculty of Production Engineering, University of Bremen, Badgasteiner Str. 1, 28359 Bremen, Germany; i.okulov@iwt.uni-bremen.de; 8 Leibniz Institute for Materials Engineering—IWT, Badgasteiner Str. 3, 28359 Bremen, Germany

**Keywords:** additive manufacturing, particle size distribution, bimodal powder, nanoparticles, Vickers microhardness

## Abstract

The particle size distribution significantly affects the material properties of the additively manufactured parts. In this work, the influence of bimodal powder containing nano- and micro-scale particles on microstructure and materials properties is studied. Moreover, to study the effect of the protective atmosphere, the test samples were additively manufactured from 316L stainless steel powder in argon and nitrogen. The samples fabricated from the bimodal powder demonstrate a finer subgrain structure, regardless of protective atmospheres and an increase in the Vickers microhardness, which is in accordance with the Hall-Petch relation. The porosity analysis revealed the deterioration in the quality of as-built parts due to the poor powder flowability. The surface roughness of fabricated samples was the same regardless of the powder feedstock materials used and protective atmospheres. The results suggest that the improvement of mechanical properties is achieved by adding a nano-dispersed fraction, which dramatically increases the total surface area, thereby contributing to the nitrogen absorption by the material.

## 1. Introduction

Additive manufacturing is becoming increasingly popular and has a high demand in various applications due to its flexible approach and excellent materials choice [1,2,3]. The laser powder bed fusion (L-PBF) technique is one of the most widely applied additive manufacturing methods [4]. It is known that the L-PBF printing parameters strongly affect the resulting material properties, such as the density, surface quality, mechanical properties, and even phase composition [2,5,6]. The laser-related settings, such as the laser power, spot size, pulse duration, or pulse frequency, can be tuned as well as the scan-related parameters, such as the scan speed, hatch spacing, and scan strategy [7,8]. Further crucial parameters are specific powder-related characteristics, such as its morphology, particle size distribution (PSD), porosity, and chemical composition [9,10]. These parameters define the rheology, which influences the powder spreading and packing density of the individual layers.

It is known that the packing density defines the layer thickness as well as its thermal conductivity, which strongly correlates with laser absorption [4]. It is established that the higher the powder packing density, the higher the bed thermal conductivity, and the better the mechanical properties of the part [4]. Jacob et al. [11] found that powders with a wide PSD increase the density of layer packing and decrease the flowability, which is crucial to powder spreading. Liu et al. [12] revealed that gas-atomized 316L steel powder with a narrow PSD provides an improved flowability, leading to a high ultimate tensile strength and robust printed components. In addition, the maximum packing density can be reached by varying the particle size ratio between the large and small particles. For instance, McGeary et al. [13] revealed that a 1:7 particle size ratio leads to the optimal packing density. The protective atmosphere (or shielding gas) and its pressure during printing also have a critical influence on the material [14,15]. It is well-known that inert atmospheres, such as argon, nitrogen, and helium, are applicable for L-PBF. For example, the use of a nitrogen atmosphere can promote nitriding of printed parts [16]. It was found that the created nitrogen ions diffuse into the surface layer of 316L steel at high temperatures, combine with metal atoms and form a solid nitrogen solution in the matrix. This leads to an increase in the microhardness, wear resistance, and corrosion resistance of materials [17,18,19,20,21,22,23]. Mukhtar et al. [24] show that nitriding as the thermo-chemical treatment can improve wear resistance and surface hardness and reduce the fatigue life of AM Ti64 compared to AM Ti64 subject to the same thermal treatment without nitriding. Klimova et al. [25] studied the effect of nitrogen (0.5–2.0 at.%) on the structure and mechanical properties of CoCrFeMnNi high entropy alloy as-cast condition. They showed that increase in the N content to 2.0 at.% resulted in the precipitation of a small amount (<1%) of the Cr-rich M_2_N nitride particles at the face-centered cubic (FCC) grain boundaries. The increase in yield strength in proportion to the N content was attributed to the solid solution hardening.

In this work, the influence of nano- and microparticles mixture, forming a bimodal powder size distribution on the obtained structural and mechanical properties of printed material is studied. Furthermore, the effect of a protective atmosphere on the printing process is demonstrated.

## 2. Materials and Methods

### 2.1. Powder Feedstock

The powder morphology was investigated by scanning electron microscopy (SEM) (Quattro S equipped with EDAX elemental analyzer, FEI Company, Hillsboro, OR, USA). The powder PSD were evaluated using the SALD-2300 laser diffraction particle size analyzer (Shimadzu Corporation, Kyoto, Japan).

**Figure 1:**
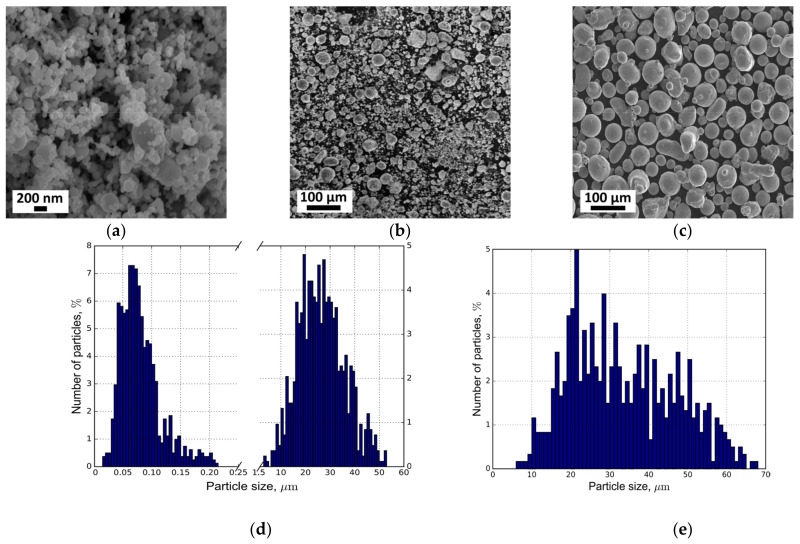
SEM images of the bimodal 316L powder consisting of a mixture of nano (**a**) and micro (**b**) scale fractions and unimodal 316L powder (**c**). PSD of nano and micro-scale fractions of the bimodal powder (**d**) and PSD of the unimodal powder (**e**).

Stainless steel 316L was used as a feedstock material. The Höganäs powder with a unimodal PSD of 20–53 µm (see Figure 1e) is compared with the originally produced powder with a bimodal PSD of 20–150 nm and 5–50 µm (see Figure 1d). The nanoscale fraction was produced using the electric explosion of wire (EEW) method without technological passivation [26]. The mass ratio of nanoparticles to microparticles was approximately 1:4. It should be noted that the microscale part of the bimodal PSD is more symmetric than the nanoscale one, which has a slowly decaying tail in the range of 100–200 nm. The chemical content of the powders is provided below in Section 3.2.

The SEM images of the both powders are shown in Figure 1. Most particles had a nearly ideal spherical shape. However, there were ellipsoidal particles and satellites present. Additionally, due to the small size, the electrostatic Coulomb force at the nano-scale was more effective. Hence, nanoaggregation was observed (Figure 1a).

The flowability testing of both powders was carried out using ISO 4490:2018(e) at room temperature. The tested portion of the dried powder weighed 50.0 ± 0.1 g.

The experiment showed that the Hall flow rate of the unimodal 316L steel powder was equal to 4 s/50 g according to the test method. In contrast, the Hall flow rate of the bimodal 316L steel powder has not exhibit flowability in the mentioned experiment, which can be explained by the high density of powder packing through the output orifice of the funnel.

### 2.2. Laser Powder Bed Fusion and Sample Preparation

**Figure 2:**
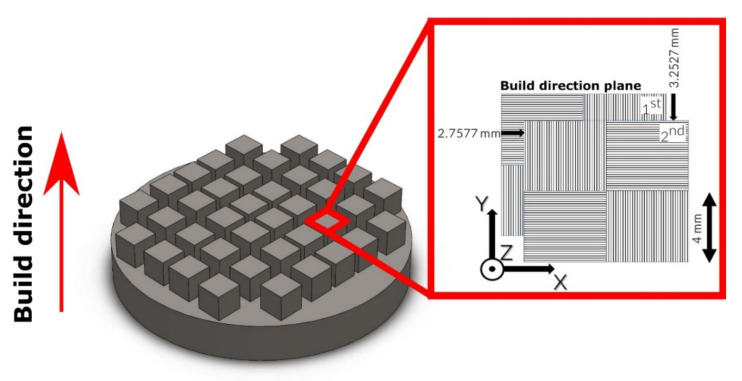
Sketch of the scanning strategy and the build direction used for the printing process.

The samples were printed via the L-PBF technique using a Trumpf TruPrint 1000 printer (TRUMPF GmbH, Ditzingen, Germany). The corresponding laser-related settings, such as the laser power (*P*), spot size (*d*), and scan-related parameters, such as the scan speed (*v*), hatch spacing (*h*), scan strategy, and other printing parameters, are shown in Table 1. The scan strategy consisted of a square pattern that was rotated 90°, forming a chess-board structure, and shifted 2.7577 mm and 3.2527 mm along the X-axis and Y-axis, respectively (Figure 2). The cubic parts with a size of 10 × 10 × 10 mm^3^ were 3D-printed in argon and nitrogen protective atmospheres at specific laser energy *E* (J mm^−3^), which was calculated as follows for the particular layer thickness (*l*):

(1) $$E=\frac{P}{hvl}$$

For the SEM analysis, the printed cubic samples were cut along and across the build direction using the Accutom-100 cutting machine (Struers, Ballerup, Denmark). The samples were then mounted via a TechPress 2 machine (Allied Corp., Rancho Dominguez, CA, USA), grounded, and polished with a diamond suspension 40 nm on a pressure-free cloth using a MetPrep 3 machine (Allied Corp.). To reveal the subgrain structure, the polished surfaces were additionally etched by HCl (50 mL)/ethanol (50 mL)/CuCl_2_ (2.5 g) solution at room temperature and dwell time of 60 s.

### 2.3. Microstructure Characterization and Analysis of Nitrogen Content

Crystallographic texture analysis was performed via electron backscatter diffraction (EBSD) using a Tescan Mira 3 LMH system (TESCAN, Brno, Czech Republic). The grain structure analysis was conducted using orientation imaging microscopy (OIM) [27]. The EBSD maps were acquired for the top and front sides of the printed samples with the scanning step size of 1 µm.

Both feedstock powders and top side of the samples 3D-printed in different protective atmospheres were analyzed with X-ray diffraction (XRD) using a Bruker D8 ADVANCE (Bruker Corporation, Billerica, MA, USA) diffractometer with CuK*α* radiation (with a wavelength of 1.5418 Å) over a 2*θ* range between 30° and 100° at room temperature. The step size and dwell time were 0.005 °C and 3 s, respectively.

The nitrogen content was determined using a LECO TC-136 nitrogen determinator (LECO Corporation, St. Joseph, MI, USA).

### 2.4. Porosity/Density Measurements

The planar porosity (normal plane to the build direction) was defined using Axio Scope. A1 optical microscopy (Carl Zeiss AG, Jena, Germany) with Thixomet Pro software (Thixomet Company, Saint Petersburg, Russia) and analyzed based on the American Society for Testing and Materials (ASTM) E1245-03.

The volumetric porosity *δ* was determined as follows:

(2) $$\delta =\frac{V_{\mathrm{voids}}}{V_{\mathrm{cub}}}=1-\frac{m_{\mathrm{mat}}^{\mathrm{air}}}{\rho_{\mathrm{mat}}V_{\mathrm{cub}}},$$

where *ρ*_mat_ is the 316L steel powder density at room temperature (7957 kg m^−3^ [28]), $m_{\mathrm{mat}}^{\mathrm{air}}$ is the weight of the cubic sample in the air (kg). *V*_cub_ is the volume that was defined using the Ohaus density determination kit (OHAUS Corporation, Parsippany, NJ, USA) and was calculated as:

(3) $$V_{\mathrm{cub}}=\alpha \frac{m_{\mathrm{mat}}^{\mathrm{air}}-m_{\mathrm{mat}}^{\mathrm{alc}}}{\rho_{\mathrm{alc}}-\rho_{\mathrm{air}}},$$

where *α* is the balance correction factor (0.99985), $m_{\mathrm{mat}}^{\mathrm{alc}}$ is the weight of the cubic sample in alcohol (kg), *ρ*_alc_ is the density of the alcohol at room temperature (788.5 kg m^−3^), and *ρ*_air_ is the density of the air at room temperature (1.2 kg m^−3^).

In addition, the density of each sample *ρ*_meas_ was determined by the Archimedes principle following the full infiltration and calculated as:

(4) $$\rho_{\mathrm{meas}}=\frac{m_{\mathrm{mat}}^{\mathrm{air}}}{m_{\mathrm{mat}}^{\mathrm{air}}-m_{\mathrm{mat}}^{\mathrm{alc}}}\left(\rho_{\mathrm{alc}}-\rho_{\mathrm{air}}\right)+\rho_{\mathrm{air}}.$$

### 2.5. Roughness and Vickers Microhardness Measurements

The roughness of the printed samples was measured using the AMETEK Taylor Hobson Surtronic Duo portable roughness tester (AMETEK, Inc., Berwyn, PA, USA) based on ISO 4287. The surface scanning direction was parallel to the build direction of the printed sample. A stylus was applied, and the diamond had a radius of 5 µm. The arithmetic mean deviation *R*_a_ was measured on the traverse length of 5 mm with a gauge force of 200 mg, and the traverse speed was 2 mm s^−1^.

The microhardness of the printed samples was measured by microindentation testing. The measurements were conducted with a Metrotest Vickers microhardness Tester ITV-1-AM (Metrotest LLC, Neftekamsk, Russia). The plane for the microindentation was normal to the build direction of the printed sample. The Vickers pyramid diamond indenter was used with an expansion angle of 136°. The microhardness measurements included six points on each sample in equivalent locations. The approximate distance between the measurement points was 300 µm, the applied load was 3 N, and the creep time was 5 s.

## 3. Results

### 3.1. Microstructural Analysis

**Figure 3:**
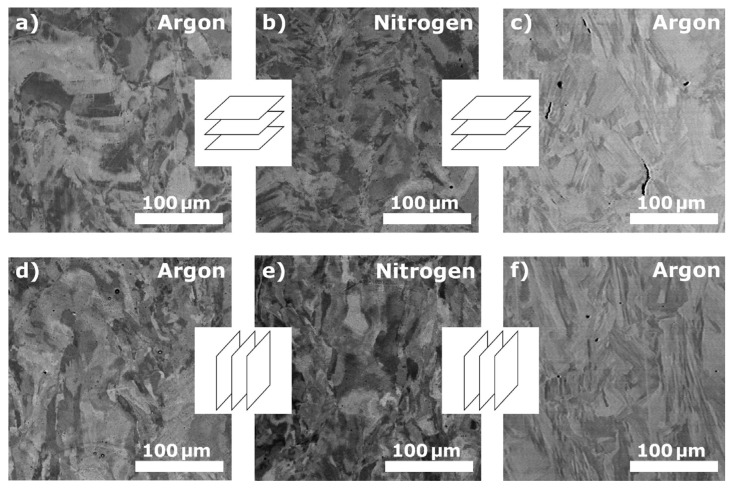
SEM images of 316L steel additively manufactured using bimodal (**a**,**b**,**d**,**e**) and unimodal (**c**,**f**) powders in different protective atmospheres (argon and nitrogen).

**Figure 4:**
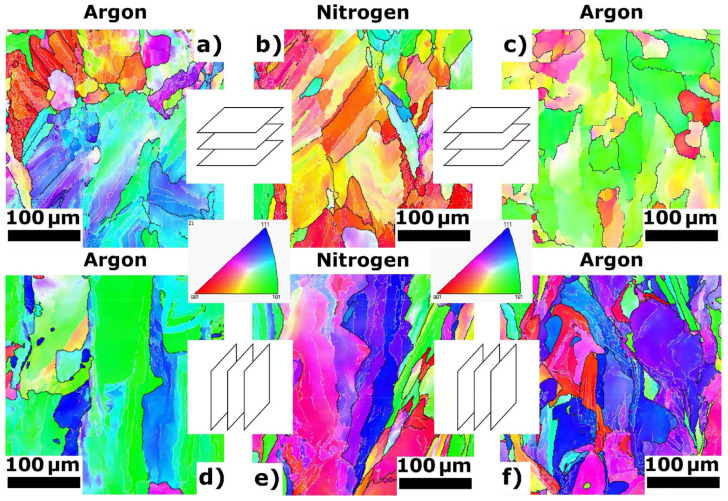
Electron backscatter diffraction (EBSD) patterns of 316L steel additively manufactured using bimodal (**a**,**b**,**d**,**e**) and unimodal (**c**,**f**) powders in different protective atmospheres (argon and nitrogen). The average grain sizes are as follows: (**a**) 34.2 µm, (**b**) 47.7 µm, (**c**) 35.2 µm, (**d**) 43.6 µm, (**e**) 56.8 µm, and (**f**) 45.6 µm.

The SEM analysis results are shown in Figure 3 for the samples with the following printing parameters: the laser power was 120 W, and the scanning speed was 300 mm s^−1^ (the resulting specific laser energy was *E* = 250 J mm^−3^). The results of the EBSD analysis and estimated average grain sizes are shown in Figure 4. In the top-down view (Figure 4a–c), the dominant crystallographic texture comprised columnar grains for both PSDs and protective atmospheres. It should be noted that predominant 〈001〉 crystallographic direction is observed only for the top-down texture of fabricated samples from the bimodal PSD in a nitrogen atmosphere (Figure 4b) [29]. In addition, other directions, such as 〈111〉 and 〈101〉, were identified for argon atmosphere, respectively in Figure 4a,c [2,30]. Different textures were recognized in the front view (Figure 4d–f). The prevailing crystallographic directions 〈101〉 and 〈111〉 are observed.

### 3.2. Phase Analysis

**Figure 5:**
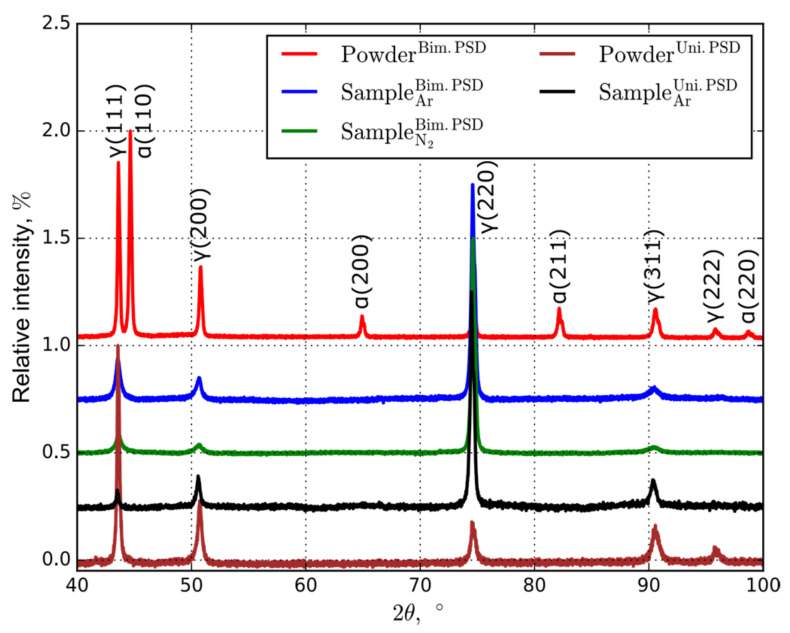
Normalized XRD patterns up to the maximum for various 316L steel powders and samples printed in different atmospheres.

The X-ray diffraction (XRD) analysis is shown in Figure 5. Depending on solidification and heat treatment conditions, the 316L steel can form two phases: the *α*-phase (ferritic matrix with body-centered cubic (BCC) lattice) and the *γ*-phase (austenitic matrix with FCC crystalline structure) [31]. The high-temperature *α*-phase is also known as *δ*-phase (delta ferrite). The bimodal powder consists of both phases (Figure 5 red line). This fact is attributed to extremely high cooling rates that occur in the production of powder by the EEW method [26]. The unimodal powder and all the samples contain the *γ*-phase only (Figure 5 brown and black lines). For samples fabricated from the bimodal powder, the *α*-phase is absent after the printing process in both atmospheres (Figure 5 blue and green lines). Therefore, the final material is fully austenitic due to quite high cooling rates in laser powder bed fusion. Under such solidification conditions, the austenite–ferrite transition in 316L steel is strongly suppressed [32]. Furthermore, for the printed samples processed in nitrogen, the doping with a small amount of nitrogen stabilizes the formation of the austenite phase [5,17,33]. For the bimodal powder, the relative intensities of the diffraction peaks for the samples 3D-printed in nitrogen are lower than those processed in argon, which can be explained by different crystallographic textures [34].

**Figure 6:**
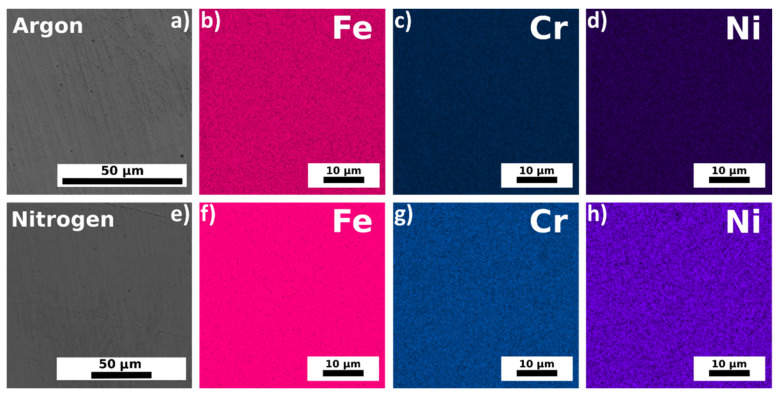
Elemental distribution maps of the additively manufactured 316L steel samples using bimodal powder. The samples were fabricated under different protective atmospheres, namely argon (**a**–**d**) and nitrogen (**e**–**h**).

The energy-dispersive X-ray (EDX) results for the samples fabricated from the bimodal powder under argon (Figure 6a) and nitrogen (Figure 6e) protective atmospheres are shown in Figure 6b–d and Figure 6f–h, respectively. The printing was conducted with a laser power of 150 W and a scanning speed of 600 mm s^−1^ (the resulting specific laser energy was *E* = 156 J mm^−^^3^).

The chemical compositions of the feedstock powders and the samples additively manufactured from them are shown in Table 2. One can see that the element contents slightly vary. The bimodal powder and the sample fabricated in argon from bimodal powder contain nitrogen about twice as high as that of the unimodal powder. Furthermore, there is no additional incorporation of nitrogen for bimodal powder during printing in a nitrogen protective atmosphere.

### 3.3. Density, Porosity, Microhardness, and Roughness

**Figure 7:**
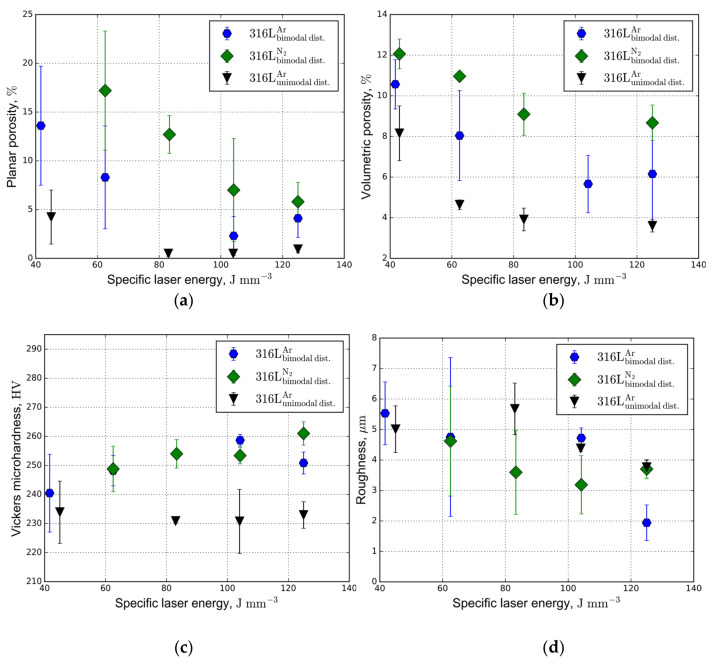
Microstructural and mechanical characteristics of the additive manufactured 316L steel samples from bimodal and unimodal powders under different protective atmospheres: (**a**) planar and (**b**) volumetric porosity, (**c**) Vickers microhardness, and (**d**) surface roughness.

**Figure 8:**
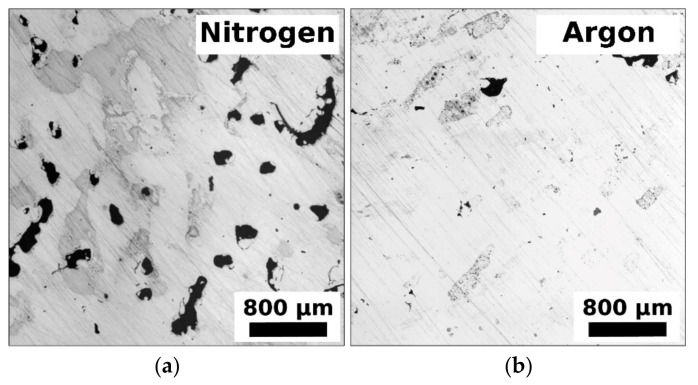
The optical images of the polished top side of the samples additively manufactured from bimodal powder in different protective atmospheres. The specific laser energy is *E* = 125 J mm^−3^.

The porosity, microindentation, and surface roughness results for various PSDs and protective atmospheres are shown in Figure 7. The best values of density, Vickers microhardness, and surface roughness at different specific laser energy are shown in Table 3. It was found that the porosity of the 316L steel samples printed from the bimodal powder was higher for the same laser-related settings than that for the samples printed from the unimodal powder (Figure 7a,b) regardless of the protective atmosphere. The porosity decreases with increasing specific laser energy, which is consistent with [35,36,37]. The high porosity at low specific laser energy can be explained by insufficient wettability and lack of complete melting of powder particles. Starting from specific laser energy of 120 J mm^−3^, the planar and volumetric porosity of the samples 3D-printed from both powder feedstock gradually increases (Figure 7a,b). For the bimodal powder, the planar porosity of the sample 3D-printed in nitrogen is ~15% higher than for argon atmosphere (Figure 8).

The primary mechanism of substantial pore formation observed for the 3D-printed samples using the bimodal powder is as follows. According to [38], the density of samples 3D-printed using a bimodal powder is typically higher than that of samples 3D-printed using a unimodal powder. However, due to the high particle size ratio of 1:1000, the opposite effect is observed (see Table 3). This is mainly due to the lower flowability of the bimodal powder. The small particles are dragged and binded with the larger particles producing agglomerates, leading to a poor flowability during the powder coating and, ultimately, causing a lower packing density and layer structure perturbation. As a result, the measured porosity of the samples is approximately 9–10%, which is twice as much as 5% porosity obtained for unimodal powder (see Table 3).

There is a noticeable dependence of the resulting porosity on the protective atmosphere (Figure 7a,b). Presumably, the possible effect can be explained as follows. Keyhole formation and its instability lead to trapping of ambient gas bubbles, causing formation of macroscopic pores [39]. The gas–liquid interface motion can be described by Newton‘s second law, which states that acceleration is inversely proportional to density. Therefore, a lower density contributes to a more intensive void formation. According to the ideal gas law, the gas density is defined as *ρ*_G_ = *pM*_G_/*RT,* where *M*_G_ is the gas molar mass, *R* is the universal gas constant, *T* is the temperature, and *p* is the external atmospheric pressure. Since the density is proportional to the molar mass under the same conditions, we can conclude that argon as a heavy gas (30% heavier than nitrogen) yields less porosity.

The Vickers microhardness results for samples fabricated with both PSDs and protective atmospheres are shown in Figure 7c. The Vickers microhardness of the samples fabricated from the bimodal powder feedstock is observed to be 10% higher than those from the unimodal one, regardless of the protective atmosphere, which is also mentioned in [38]. Moreover, for bimodal powder, the average Vickers microhardness of the printed samples processed in the nitrogen was slightly higher than that for samples processed in the argon, which is in agreement with the results obtained in [15].

**Figure 9:**
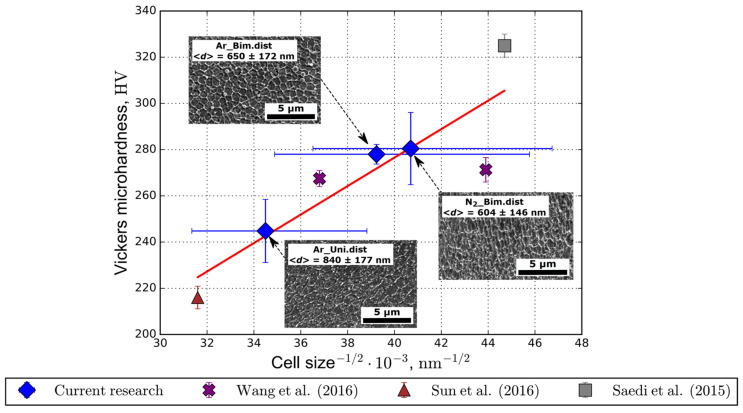
Vickers microhardness as the function of average cell size 〈dd〉 for different powder PSDs and protective atmospheres [8,41,42].

The SEM analysis of etched polished surfaces normal to the build direction reveals the cellular structure. Based on the average cell size estimated from the SEM images, the samples fabricated from the bimodal powder have a smaller cell spacing (Figure 9). This finding can be attributed to higher cooling rates [40] and the presence of nitrogen in the powder feedstock [15]. Thus, according to the Hall–Petch relation, the Vickers microhardness of 3D-printed samples from the bimodal powder is observed to be higher (Figure 7c).

Assumedly, the presence of high nitrogen content in the powder is the result of the environmental influence after the end of the production method and is defined as follows. Smaller powder particles result in a higher surface area that should increase ambient gas accumulation on the powder surface due to a physical (van der Waals force) and chemical adsorption. Adsorbed nitrogen is absorbed into the metal matrix as the interstitial element and further causes the lattice distortion, leading to increased yield strength [43]. The nitrogen as the protective atmosphere can also contribute to additional hardening that is nitriding of steels [44,45]. Due to the high affinity of Cr and N, the formation of Cr_2_N nanoprecipitates is more likely compared to other metal elements [46,47]. However, according to the XRD analysis (Figure 5), we did not reveal the shift of peaks that should illustrate the presence of nitrogen in the lattice [48,49].

The surface roughness of the printed samples from the unimodal powder were slightly higher than those for the printed samples from the bimodal powder (Figure 7d). Roughness reduction was observed with an increase in the specific laser energy due to more extensive remelting of newly solidified particles, which eliminated micropores [50]. Moreover, it was established that fine particles provide a smoother surface area and absorbed the specific laser energy more efficiently than coarse particles at the same absorption index [12,51,52].

## 4. Conclusions

This work illustrates the effect of increasing the microhardness of 316L steel samples additively manufactured from a bimodal powder produced by the electrical explosion of wires method. The side effect is that the residual porosity increases due to poor flowability and bad formability of the bimodal powder. The surface roughness of fabricated samples was the same regardless of the powder feedstock materials used and protective atmospheres. Notably, it was demonstrated that regardless of the protective atmosphere, the Vickers microhardness becomes about 10% higher for samples fabricated from the bimodal powder than from the unimodal one. The average cell size decrease verifies this enhancement according to the Hall-Petch relation. The nitrogen content results determined that bimodal powder contains twice as much nitrogen as in unimodal powder due to the increase in the total surface area. Ultimately, we conclude that the increased hardness observed for bimodal powder is achieved by reduction of the average cell size and high nitrogen content. Based on these observations, the presented approach can be applied as an additional method of hardening additively manufactured parts.

## Figures and Tables

**Table 1:** The printing parameters.

Printing Parameter	Value (s)	Unit
Laser power	60–150	W
Spot size	55	µm
Scan speed	100–3000	mm s^−1^
Hatch spacing	80	µm
Layer thickness	20	µm
Square pattern side	4	mm
Gas flow (Ar/N_2_)	2.5	m s^−1^
Oxygen level	<0.3	at.%
Pressure in chamber	0.1	MPa

**Table 2:** Energy-dispersive X-ray (EDX) elemental analysis and the nitrogen content for 316L steel powders and additively manufactured samples for various PSDs and protective atmospheres.

Element	Weight [%]				
	${\mathrm{Powder}}_{}^{\mathrm{Bim}.\mathrm{PSD}}$	${\mathrm{Powder}}_{}^{\mathrm{Uni}.\mathrm{PSD}}$	${\mathrm{Sample}}_{\mathrm{Ar}}^{\mathrm{Bim}.\mathrm{PSD}}$	${\mathrm{Sample}}_{N2}^{\mathrm{Bim}.\mathrm{PSD}}$	${\mathrm{Sample}}_{\mathrm{Ar}}^{\mathrm{Uni}.\mathrm{PSD}}$
Fe	64.9	66.1	64.7	64.2	66.2
Cr	17.8	16.6	16.8	16.9	16.5
Ni	12.5	12.5	12.3	12.4	12.1
Mo	1.2	2.5	2.2	2.8	2.4
Mn	2.0	1.5	1.8	1.7	1.5
Si	0.6	0.7	0.4	0.3	0.6
N	0.127	0.068	0.122	0.096	0.067

**Table 3:** The best values of density, Vickers microhardness, and surface roughness for different PSDs and protective atmospheres.

Physical Property, Unit	PSD/Protective Atmosphere		
	Bimodal/Ar	Bimodal/N_2_	Unimodal/Ar
Density *, kg m*^−^*^3^	7280 ± 230	7190 ± 210	7520 ± 210
Vickers microhardness, HV	259 ± 2	261 ± 4	234 ± 11
Roughness, µm	1.9 ± 0.6	3.2 ± 0.9	3.8 ± 0.2

* Note: To estimate volumetric porosity, the obtained density is compared with the alloy density of 7957 kg·m^−3^ [28].

## Data Availability

No new data were created or analyzed in this study. Data sharing is not applicable to this article.
